# An overview of the role of the gut microbiota in rheumatoid arthritis

**DOI:** 10.20517/mrr.2025.69

**Published:** 2026-03-09

**Authors:** Anastasia V. Poznyak, Aleksey A. Vatlin, Vsevolod V. Pavshintsev, Nikita A. Mitkin, Olga N. Maltseva, Aleksandra S. Utkina, Alexander N. Orekhov

**Affiliations:** ^1^Institute for Atherosclerosis Research, Moscow 121609, Russia.; ^2^Institute of Ecology, Рeoples’ Friendship University of Russia (RUDN University), Moscow 117198, Russia.; ^3^Institute of Experimental Medicine, Saint Petersburg 197022, Russia.; ^4^Department of Commodity Expertise and Customs Business, Plekhanov Russian University of Economics, Moscow 115054, Russia.

**Keywords:** Rheumatoid arthritis, autoimmunity, inflammation, microbiome

## Abstract

Rheumatoid arthritis (RA) is a chronic autoimmune disease preceded by a prolonged preclinical phase marked by the emergence of autoantibodies and mucosal immune dysregulation. Evidence from human studies and animal models consistently demonstrates that gut microbiota dysbiosis contributes to this transition, particularly through impaired intestinal barrier function, activation of pro-inflammatory pathways, and molecular mimicry. Specific taxa - including *Prevotella copri*, *Collinsella aerofaciens*, and reductions in butyrate-producing bacteria - have been linked to heightened systemic inflammation, increased T helper 17 responses, and the generation of RA-associated autoantibodies. Current research also indicates that anti-rheumatic medications such as methotrexate, sulfasalazine, and minocycline produce measurable shifts in gut microbial composition, suggesting that microbiota-drug interactions may influence treatment response. Therapeutic approaches aimed at modifying gut ecology - including dietary interventions, prebiotics, probiotics, and fecal microbiota transplantation - show early potential in restoring microbial balance, improving intestinal barrier integrity, and reducing inflammatory markers, although evidence in the preclinical RA stage remains limited. Additionally, emerging data highlight the importance of intestinal autophagy and microRNA networks in regulating epithelial integrity and systemic immune activation. Taken together, the literature supports a mechanistic link between gut dysbiosis and the onset of RA. It points to microbiota-targeted strategies as promising avenues for delaying or preventing disease progression. Future studies should prioritize longitudinal analyses and interventional trials focusing specifically on individuals at risk for RA.

## INTRODUCTION

Rheumatoid arthritis (RA) is a chronic autoimmune disorder characterized by polyarthritis, leading to damage of cartilage and bone and significantly impairing an individual’s mobility. The progression of RA begins with an initial susceptibility phase influenced by genetic factors, followed by a preclinical stage marked by a breakdown in immunological tolerance, ultimately resulting in the onset of clinical arthritis^[1,2]^.

The primary objectives in treating RA are to achieve long-term remission or maintain low disease activity, prevent joint damage and disability, and reduce the risk of complications. Current pharmacological options for RA include glucocorticoids, non-steroidal anti-inflammatory drugs (NSAIDs), disease-modifying anti-rheumatic drugs (DMARDs), and small molecule inhibitors^[3-5]^. Despite the advancement of treatment strategies such as treat-to-target, the application of DMARDs, and early intervention, more than 60% of RA patients do not reach true remission following the onset of clinical arthritis. Initiating treatment during the preclinical phase of RA, before the onset of clinical symptoms, could prevent substantial joint damage and foster sustained remission. This indicates that the preclinical stage may represent a critical window for effective intervention^[6-9]^. It is important to note that remission rates vary across patient populations. Several studies indicate that certain subgroups - such as individuals with early-onset disease, seropositive patients, smokers, older adults, and some ethnic minorities - exhibit lower response rates to conventional and biologic therapies. Women also tend to have lower rates of sustained remission compared to men, likely due to differences in immune regulation and genetic background^[10]^. These findings suggest that the commonly cited 60% non-remission rate represents an overall average and may obscure poorer outcomes in specific high-risk subgroups^[11]^.

Preclinical RA is characterized by the presence of autoantibodies - such as those targeting citrullinated proteins and rheumatoid factors - resulting from a loss of immunological tolerance^[12-14]^. However, it is important to note that the definition of ‘pre-clinical RA’ is not fully standardized. Different studies and guidelines apply varying criteria - such as seropositivity for anti-citrullinated protein antibodies (ACPAs) or Rheumatoid Factor (RF), genetic risk factors, musculoskeletal symptoms without clinical arthritis, or subclinical synovitis on imaging - resulting in heterogeneity across cohorts^[15]^. This ambiguity complicates the identification of truly at-risk individuals and may influence both the timing and interpretation of preventive intervention strategies.

Emerging evidence from human cohorts and experimental models indicates that the gut microbiota contributes to the transition from preclinical to clinically manifest RA. Microbial dysbiosis has been implicated in the breakdown of immunological self-tolerance and in the amplification of inflammatory pathways through mechanisms such as molecular mimicry^[16-19]^. Bacterial peptides that share structural similarity with established RA autoepitopes may promote autoantibody generation through cross-reactive immune responses, thereby contributing to joint tissue damage. Integrated microbiome and metabolomics studies have further demonstrated that microbially derived metabolites act as immunomodulatory signals that influence immune cell differentiation and effector functions^[20-22]^. In addition, microbiota-driven alterations in intestinal barrier integrity during the preclinical phase may facilitate trafficking of immune cells from the gut to synovial tissues, promoting disease progression^[23]^.

Lipopolysaccharide (LPS), present in the cell walls of beneficial gram-negative gut bacteria, is known to activate cytokine cascades and can drive T-cell-mediated arthritis development. However, LPS from specific bacterial strains can also produce beneficial effects on immune responses. These beneficial effects include improvements in systemic inflammation, enhanced endothelial function, reductions in oxidative stress, and modulation of metabolic pathways, collectively contributing to reduced cardiovascular risk^[24]^. Additionally, the interplay between gut microbiota, microRNAs (miRNAs), human leukocyte antigen (HLA) genes, and intestinal autophagy is crucial in regulating both local and systemic inflammation^[25-27]^.

The significant role of gut microbiota in RA development suggests that targeting it during the preclinical stage could offer novel therapeutic options. There has been considerable research into interventions that modify the intestinal microbiota for RA management, including probiotics, prebiotics, dietary modifications, antibiotics, fecal microbiota transplantation (FMT), and natural herbal products^[28-30]^. However, most investigations have primarily focused on the effects of these treatments in individuals already diagnosed with clinical RA and in animal models, rather than targeting the preclinical stage. This underscores the need for further studies to evaluate the efficacy of these interventions during the preclinical phase, aiming to prevent the onset of RA^[31-33]^.

## PRIMARY COMPONENTS OF GUT MICROBIOME

Specific gut microbial taxa have been implicated in RA pathogenesis through defined immunological and metabolic mechanisms, with corresponding therapeutic strategies proposed [Table 1].

**Table 1:** Role of gut microbiota in RA

Bacterial species	Impact on RA	Mechanism	Potential therapeutic approaches	References
*Prevotella copri*	Increased in early RA patients; associated with inflammation	Triggers inflammatory responses; molecular mimicry	Probiotics, dietary modifications	[34-54]
*Collinsella aerofaciens*	Enhances intestinal permeability; exacerbates arthritis	Compromises tight junctions; elevates inflammatory markers	Prebiotics, FMT	[55-66]
*Lactobacillaceae*	Relationship to severity varies; decreases in long-standing RA	Activates Th17 cells; potential anti-inflammatory effects	Probiotic supplementation	[72-82]
Butyrate-producing bacteria	Promotes gut health; reduces inflammation	Regulates immune responses; strengthens intestinal barrier	High-fiber diet, SCFA supplementation	[64-71]

RA: Rheumatoid arthritis; Th17: T helper 17; FMT: fecal microbiota transplantation; SCFA: short-chain fatty acid.

### Prevotella copri

Numerous studies have established a connection between RA and imbalances in the gut microbiome, though results can vary across different investigations. A notable finding is the increased abundance of the *Prevotella* genus, particularly *Prevotella copri *(*P. copri*), in individuals with early-stage RA compared to those without the condition^[34,35]^. Interestingly, this overrepresentation of *P. copri* is not found in patients who have undergone treatment for RA. Advanced metagenome-wide shotgun sequencing has revealed that RA patients also show increased levels of various other *Prevotella* species beyond *P. copri*^[36,37]^.

The contribution of *P. copri* to RA development is believed to involve the induction of inflammatory responses and the phenomenon of molecular mimicry, where microbial proteins resemble host proteins. As mentioned earlier in this review, the introduction of *P. copri* into germ-free SKG mice resulted in the development of arthritis and elevated levels of Th17, interleukin (IL)-23, and IL-1^[38,39]^. Similarly, SKG mice with a microbiota rich in *Prevotella* from RA patients exhibited worsened arthritis symptoms, accompanied by increased Th17 cells and related cytokines. Collectively, these findings suggest that an imbalanced gut microbiome, particularly characterized by species of *Prevotella* - especially *P. copri* - may precede and promote the onset of arthritis^[40-42]^.

The inflammatory role of *P. copri* extends beyond murine studies. Evidence shows that a peptide derived from *P. copri* can bind to human leukocyte antigen – DR isotype (HLA-DR) molecules, triggering Th1-type inflammatory responses in early RA patients. Both Immunoglobulin A (IgA) and Immunoglobulin G (IgG) antibodies against *P. copri* have been identified in patients at both early and established stages of the disease^[43,44]^. Additionally, there appears to be a correlation between antibodies against *P. copri*, levels of Th17 cytokines, and ACPAs. Furthermore, the 16S ribosomal RNA (16S rRNA) of *P. copri* has been detected in the synovial fluid of some patients, suggesting that the gut microbiome may contribute to autoimmune responses affecting the joints through microbial peptides^[45]^. However, a direct link between intestinal levels of *P. copri* and its proteins has not yet been established. Nevertheless, evidence for a causal relationship remains limited; most available findings are observational and demonstrate association rather than mechanistic proof. Only a few experimental studies using microbiota transfer into germ-free mice suggest a contributory - but not definitive - causal role for *P. copri* in promoting inflammatory responses^[46]^.

Interestingly, *P. copri* antigens share structural similarities with N-acetylglucosamine-6-sulfatase, an autoantigen associated with RA that triggers T- and B-cell responses in approximately half of RA patients. It is hypothesized that in genetically predisposed individuals, the immune system recognition of *Prevotella*-derived epitopes might lead to T-cell activation in the gut, which could then migrate to the joints, suggesting a possible mechanism for RA pathogenesis through molecular mimicry^[47,48]^.

Nevertheless, some *Prevotella* species, such as *Prevotella histicola *(*P. histicola*), have been found to reduce the severity of arthritis in murine models, and the *Prevotella* genus is one of the most abundant groups of beneficial commensal bacteria in healthy individuals. These conflicting findings highlight the intricate interplay between various bacterial species and genetic factors in the development of RA. More comprehensive longitudinal studies are needed to fully elucidate the role of *Prevotella* species in autoantibody production and the pathogenesis of RA^[49-51]^.

Although *P. copri* has frequently been associated with a pro-inflammatory profile in early RA, emerging evidence shows that not all *Prevotella* species exert similar effects. Mechanistic studies indicate that strain-level differences within *P. copri *influence immunogenicity, including variation in carbohydrate-active enzymes, LPS structure, and antigen presentation pathways^[52]^. Some strains promote Th17-mediated inflammation, whereas others appear to support mucosal immune tolerance. In contrast, *P. histicola* has been shown to induce regulatory T cells (Tregs), enhance intestinal barrier integrity, and suppress pro-inflammatory cytokines, resulting in protection against arthritis in murine models^[53]^. Comparative genomic and functional analyses confirm that distinct *Prevotella *species - and even different strains within a species - possess divergent metabolic capacities and immunomodulatory properties. These findings suggest that the ‘dual effect’ reflects underlying functional heterogeneity across *Prevotella* taxa rather than a paradoxical behavior of a single organism^[54]^.

### Collinsella

Several bacterial genera, including *Collinsella*, *Lactobacillaceae*, *Eggerthella*, and *Actinomyces*, have been associated with RA, suggesting that the disease development results from complex interactions among various bacterial species, genetic factors, and environmental influences. Notably, *Collinsella*, especially the species *Collinsella aerofaciens *(*C. Aerofaciens*), has been shown to increase intestinal permeability in RA mouse models by reducing the expression of proteins that form tight junctions^[55-57]^. However, the evidence supporting a causal role for *Collinsella* in intestinal permeability and inflammation remains limited. Much of the current literature is based on correlations between increased *Collinsella* abundance and inflammatory markers. Experimental support comes primarily from a single mechanistic study showing that *C. Aerofaciens* reduced tight junction protein expression and increased IL-17A production *in vitro*, suggesting a potential causal effect^[58]^.

Yet, alternative interpretations exist: *Collinsella* may increase in abundance because it can utilize Maillard reaction products (MRPs) and advanced glycation end products (AGEs), which are themselves pro-inflammatory^[59]^. MRPs and AGEs accumulate in inflammatory and metabolically stressed environments and are strongly associated with oxidative stress, immune activation, and epithelial dysfunction. Several gut microbial taxa, including members of the Actinobacteria phylum to which* Collinsella* belongs, possess metabolic capacities that enable the utilization of complex glycated substrates^[60,61]^*.* In this context, enrichment of* Collinsella* may represent a secondary ecological response to increased availability of MRPs and AGEs rather than a primary pathogenic driver of inflammation^[62]^*.* In such a scenario, *Collinsella *expansion could reflect an adaptive or compensatory response rather than a driver of disease.

Importantly, direct in vivo evidence demonstrating that* Collinsella *independently initiates intestinal barrier disruption or systemic inflammation in humans is currently lacking. To date, evidence that* C. Aerofaciens *drives intestinal barrier loss in humans is indirect and largely inferential, relying on associations rather than causative demonstrations^[63]^*. *Mechanistic evidence that *Collinsella* causes permeability defects primarily comes from *in vitro* and animal experiments (e.g., reduced tight-junction protein expression and increased IL-17A after exposure to *Collinsella* or its products)^[64,65]^. Human studies, by contrast, report associations between increased *Collinsella* abundance and RA or with biomarkers of gut barrier disruption (e.g., elevated zonulin, LPS/LPS-binding protein, and soluble CD14), but these are observational and cannot establish causality. Importantly, experimental targeting of intestinal barrier regulators such as zonulin has demonstrated that barrier dysfunction can precipitate arthritis in murine models, supporting the biological plausibility of a gut → joint pathway. However, this still does not prove that expansion of *Collinsella* is a primary initiating event in humans^[66]^.

In short, the current human evidence links *Collinsella* to RA and to indirect markers of permeability; however, direct, longitudinal or interventional human data demonstrating that *Collinsella* alone initiates barrier breakdown and subsequent RA onset are lacking. This bacterium elevates levels of inflammatory chemokines such as IL-17A, C-X-C motif chemokine ligand 1 (CXCL1), C-X-C motif chemokine ligand 5 (CXCL5), and nuclear factor of kappa light polypeptide gene enhancer in B-cells 1 (NF-κB1), exacerbating both the incidence and severity of arthritis. These findings indicate that increased intestinal permeability may be a crucial mechanism through which gut dysbiosis affects RA^[67]^.

The proliferation of *C. Aerofaciens* is believed to induce intestinal inflammation and compromise the integrity of the epithelial barrier, allowing bacterial antigens to enter the bloodstream^[68-70]^. This translocation can trigger immune responses at distant sites, including the joints. Additionally, studies have identified a reduction in the genera *Roseburia *and *Faecalibacterium* in individuals with RA. These bacteria are known for their butyrate production and anti-inflammatory properties, playing a vital role in maintaining intestinal barrier health. This supports the hypothesis that a compromised intestinal barrier contributes to the onset of RA and suggests potential avenues for treatment.

However, interpretation of these findings is complicated by the compositional nature of most high-throughput microbiome datasets. Because sequencing-based approaches primarily generate relative abundance data, apparent decreases in butyrate-producing genera such as* Roseburia *and* Faecalibacterium *may not reflect true biological depletion but instead result from proportional shifts driven by expansion of other taxa, including* Collinsella *or* Prevotella. *Without direct assessment of total bacterial load, it is not possible to distinguish genuine microbial loss from compositional artifacts^[71,72]^*. *Relative shifts in genera such as *Roseburia* and *Faecalibacterium* may therefore not necessarily reflect true increases or decreases in their absolute abundance, but rather proportional changes resulting from alterations in other community members.

Several methodological approaches have been proposed to overcome these limitations, including quantitative PCR-based normalization, flow cytometry-assisted microbial counting, and the use of spike-in standards to estimate absolute microbial abundances. While these methods improve biological interpretability and cross-study comparability, they remain underutilized in RA research due to increased technical complexity and cost^[73,74]^. Without direct quantification of total bacterial load, it is not possible to distinguish genuine biological expansions or contractions from compositional artifacts. Integration of absolute quantification approaches, such as quantitative PCR-based normalization or spike-in standards, would be required to clarify the direct relationship between these taxa and RA^[75,76]^.

In collagen-induced arthritis (CIA) mouse models, butyrate has been demonstrated to alleviate arthritis symptoms. However, its ability to activate IL-23 and promote Th17 cell differentiation also raises the possibility that butyrate may contribute to RA development. Further investigation is needed to establish the significance of intestinal integrity as a therapeutic target for RA and to understand the effects of butyrate-producing bacteria on the disease^[77-79]^.

#### Lactobacillaceae

The growth of *Lactobacillaceae* has been observed in patients with early and established RA; however, a decrease in *Lactobacillaceae* levels occurs in individuals with long-standing RA. Notably, *Ligilactobacillus salivarius* (*L. salivarius*), present in both the oral cavity and intestines, has been associated with disease severity^[80,81]^. This connection between *Lactobacillaceae* and RA is supported by findings from animal studies. For example, the introduction of *L. bifidus* into IL-1ra-/- mice was sufficient to induce arthritis, and an increased abundance of *Lactobacillaceae* was detected in CIA mice before the onset of arthritis. Similar to *Prevotella*, *Lactobacillaceae* are believed to contribute to RA development by promoting Th17 cell activity and associated cytokines, as well as activating Th1-cell responses^[82-85]^.

Despite this association, various studies in both animals and humans have shown that *Lactobacillaceae* consumption can alleviate arthritis symptoms and reduce inflammation. For instance, treatment with *Lacticaseibacillus casei* in rats has been reported to restore gut dysbiosis, diminish arthritis severity, and lower pro-inflammatory cytokine levels. Moreover, both *L. salivarius *and *Lactobacillus plantarum *have been found to decrease Th17 cells while increasing Tregs in CIA mice, resulting in milder arthritis. A comprehensive review concluded that while *Lactobacillaceae* supplementation reduces IL-6 levels, it does not significantly impact arthritis in humans. Therefore, the implications of findings related to *Lactobacillaceae* warrant caution, and further research is necessary, particularly given the widespread use of *Lactobacillaceae* species as probiotics^[86-88]^.

The apparently contradictory effects of *Lactobacillaceae* taxa in RA are best explained by strain-specific functional differences and strong dependence on the host microenvironment. Experimental studies indicate that certain taxa enriched in untreated RA - often reported at the species level (e.g., *L. salivarius*) rather than as fully characterized strains - can promote Th17-associated immune responses and exacerbate inflammatory pathology. In contrast, well-characterized probiotic strains, including *Lacticaseibacillus rhamnosus* (*L. rhamnosus*) *GG*, *Lacticaseibacillus paracasei Shirota strain* (*LcS*), *Lactobacillus helveticus* R0052, and *Limosilactobacillus fermentum ME-3*, have been shown to enhance regulatory T-cell responses, improve epithelial barrier integrity, and increase short-chain fatty acid (SCFA) production in experimental systems. These divergent effects highlight that functional outcomes cannot be inferred at the genus level. Instead, biological activity is highly dependent on strain-level genomic and metabolic properties and their interaction with host immune networks. Rigorous strain-level characterization is therefore essential for interpreting reported effects and for the rational design of microbiota-based interventions in RA^[63,89]^.

## TRANSFORMING THE GUT-JOINT AXIS

Multiple microbiota-targeted interventions, including dietary modification, probiotics, FMT, and miRNA-based approaches, have been investigated for their potential roles in preclinical RA, with proposed mechanisms of action, current evidence, and research gaps summarized in Table 2.

**Table 2:** Therapeutic strategies targeting gut microbiota in preclinical RA

**Intervention**	**Mechanism**	**Evidence**	**Future directions**	**References**
Dietary changes	Modulates gut microbiota composition	Mediterranean and vegetarian diets reduce RA activity	Investigate long-term effects and specific dietary components	[116-119,124-128]
Probiotics	Restores microflora balance	Mixed results; some studies show symptom relief	Explore specific strains beneficial for RA prevention	[78-80,116-118]
FMT	Restores gut microbiome from healthy donors	Show promise in animal models; variable human results	Protocol standardization and long-term safety assessments	[144-156]
MicroRNA modulation	Alters gut immune responses	Emerging research linking miRNAs to microbiome composition	Clinical trials to assess efficacy in RA prevention	[180-202]

RA: Rheumatoid arthritis; FMT: fecal microbiota transplantation.

### Anti-rheumatic drugs and the gut microbiota

The alterations in the gut microbiome associated with RA, both in terms of composition and abundance, prompt an investigation into how existing RA treatments affect patients’ gut bacteria. Anti-rheumatic medications, including chemical agents, can disrupt microbial balance by influencing immune responses and directly interacting with microbes as foreign entities^[90-92]^. Minocycline, an antibiotic from the tetracycline class, has been used as a DMARD for RA treatment and continues to be prescribed for a limited number of patients in certain areas. The rationale for using antibiotics in RA was initially based on the hypothesis of an infectious cause, particularly targeting Mycoplasma. However, the therapeutic effects of minocycline in RA are now attributed to its anti-inflammatory and immune-modulating properties rather than its antimicrobial activity^[93,94]^.

As research progresses in understanding the role of gut and other microbiota in inflammatory arthritis, there is increasing interest in how tetracyclines such as minocycline might influence RA through changes to the gut microbiome. Although direct evidence of minocycline’s effect on gut microbiota is limited, studies indicate that a single dose can significantly alter fecal microbiota composition in healthy individuals. This observation raises the possibility that minocycline’s therapeutic effects in RA are mediated, at least in part, via shifts in the gut microbiota that alter host immunity or microbial metabolite profiles. Antibiotic-induced decreases in taxa linked to pro-inflammatory phenotypes (e.g., some *Actinobacteria* including *Collinsella*) and concurrent changes in SCFA producers could plausibly reduce systemic inflammation and modify drug pharmacokinetics^[95]^. However, the evidence is currently indirect: most human data are short-term and descriptive. Therefore, the contribution of microbiota modulation to minocycline’s clinical efficacy remains a testable hypothesis rather than a proven mechanism. Importantly, long-term antibiotic exposure could also induce persistent dysbiosis, select for antibiotic resistance genes, or impair beneficial microbial functions, which underscores the need to evaluate chronic effects in RA populations^[96]^. Notably, minocycline treatment has been associated with a reduction in certain gut bacteria linked to RA, such as *Actinobacteria* (including *Collinsella* species) and some *Firmicutes*. Conversely, Bacteroides species, which earlier studies suggested might decrease in RA patients, were found to increase in healthy individuals following minocycline treatment^[97,98]^.

In a comprehensive analysis of fecal, dental, and salivary samples from individuals with and without RA, researchers found distinct microbiomes in RA patients compared to healthy participants, consistent with previous findings. However, this difference diminished after three months of methotrexate treatment^[99-101]^. Interestingly, a predictive model utilizing gene expression data from the microbiome organized into metagenomic linkage groups proved effective in distinguishing RA patients who responded positively to DMARD therapy from those who did not. A focused study involving 42 RA patients and 10 healthy controls, using 16S rRNA sequencing to analyze fecal microbiomes, indicated that methotrexate treatment reduced the presence of *Enterobacteriales*^[102,103]^. This effect may result from methotrexate’s interaction with the bacterial enzyme dihydrofolate reductase, which is crucial for bacterial survival, suggesting that methotrexate can unintentionally alter bacterial populations. The relationship between methotrexate and the gut microbiota is becoming increasingly prominent, as emerging evidence suggests that gut microbiota may play a significant role in methotrexate metabolism and pharmacokinetics, thereby influencing RA treatment efficacy^[104-106]^.

Sulfasalazine exhibits both antimicrobial and anti-inflammatory properties, but its effectiveness relies on activation by the gut microbiota. In its inactive form, sulfasalazine is converted into the active compound 5-aminosalicylic acid by the bacterial enzyme azuroreductase, produced by microbes in the distal gut^[107,108]^. Nevertheless, research on the impact of sulfasalazine on gut microbiota in RA remains limited. Before the advent of next-generation sequencing, one study observed significant reductions in fecal levels of *Clostridium perfringens* and *Escherichia coli* (*E. coli*) during sulfasalazine treatment. This reduction in *E. coli* was confirmed by subsequent research, which also noted a concurrent decrease in Bacteroides spp. and an increase in *Bacillus spp*.^[109,110]^. Furthermore, treatment with hydroxychloroquine, an anti-rheumatic medication, was associated with increased bacterial richness and diversity in the gut, as shown in a study utilizing 16S rRNA sequencing. This treatment appeared to enhance levels of *Faecalibacterium spp*., known for producing butyrate, a SCFA that helps reduce inflammation and regulate the intestinal barrier^[111,112]^.

While tumor necrosis factor (TNF) inhibitors are a cornerstone of RA treatment, research on their effects on the microbiome is lacking. For instance, treatment with etanercept in RA patients resulted in an increase in *Cyanobacteria* and *Nostocophycideae*, alongside a decrease in *Clostridiaceae *and *Deltaproteobacteria* within their fecal microbiomes^[113,114]^. Conversely, in CIA mice, etanercept treatment was associated with decreased gut microbial richness and diversity, characterized by an increase in *Escherichia* and *Shigella spp*., and a decrease in *Lactobacillaceae*, *Clostridium* cluster XIVa, and *Tannerella spp.* Further research is warranted to explore the effects of cytokine inhibitors on the gut microbiota in RA. However, it is essential to approach the application of findings from other conditions, such as inflammatory bowel disease (IBD), to RA with caution. Despite some shared dysbiosis across various immune-mediated inflammatory diseases, unique microbiome alterations have been observed in each condition^[115-117]^. This suggests that extrapolating microbiome findings from other immune-mediated inflammatory diseases to RA may be misleading. Disease-specific microbial alterations should be carefully characterized in RA, ideally in longitudinal studies and across diverse patient populations, to ensure that interventions target mechanisms truly relevant to RA pathogenesis rather than generic dysbiosis patterns observed in other conditions^[118]^.

### Dietary alternatives

The notion that current treatments for RA might also influence gut bacteria suggests that manipulating the gut microbiome could have therapeutic benefits for RA patients. However, findings from a French cohort challenge this idea, revealing that rigorous oral hygiene does not affect disease activity in patients with established RA, although this approach has yet to be tested in individuals with early-stage RA^[119,120]^. The relationship between gut dysbiosis and the intestinal immune system is especially critical during the early phases of inflammation, which, once initiated, may not be reversible through approaches targeting dysbiosis. Investigating modifications of dysbiosis in the early or preclinical stages of the disease, or among individuals at high risk for RA, could significantly enhance our understanding of its role in the onset of the disease^[121,122]^.

Several randomized controlled trials have assessed the impact of probiotics on RA by examining disease activity and cytokine profiles. However, a meta-analysis of four trials found no significant benefit in using probiotics as a supplementary treatment for RA. This conclusion is tentative due to the small sample sizes and varying study designs. Among the dietary interventions studied in RA, only the Mediterranean and vegetarian diets have shown a reduction in disease activity^[123-125]^. However, the long-term effects and patient adherence to these dietary interventions remain unclear. Further research is needed to explore strategies for optimizing these diets, enhancing patient compliance, and assessing their sustainability and impact on long-term disease outcomes in RA^[126]^.

SCFAs, such as butyrate, produced through the fermentation of dietary fibers by gut bacteria, play an essential role in modulating the immune system. They exert their influence on immune cells primarily through direct interaction with FFAR2 receptors located on colonic T cells and type 3 innate lymphoid cells (ILC3s). In mouse models, the absence of FFAR2 is associated with a pro-inflammatory gut state, increasing susceptibility to inflammation and infections^[127,128]^. SCFAs and FFAR2-activating compounds enhance the proliferation of ILC3s and the secretion of IL-22 in mice, providing local protection against colonic inflammation. Similarly, SCFA activation of free fatty acid receptor 2 (FFAR2) in T cells promotes the expansion of regulatory Treg cells both locally and systemically. The therapeutic potential of SCFAs was also investigated in CIA mice, where they were found to alleviate arthritis severity by modulating IL-10 levels^[129,130]^.

Fiber serves as the primary substrate for SCFA production by the gut microbiota. Given the established role of SCFAs in maintaining gut health and their potential to reduce systemic inflammation in RA, it is reasonable to propose that a fiber-rich diet could protect the gut and lower systemic inflammation in RA patients^[131,132]^. Additionally, fiber may play a significant role in the health benefits associated with vegetarian and Mediterranean diets, both known for their high fiber content. Supporting this hypothesis, a pilot study involving 36 RA patients provided a high-fiber diet supplement for 28 days. Post-treatment blood tests indicated an increase in circulating Treg cells, an improved T helper 1 (TH1) to T helper 17 (TH17) cell ratio, a reduction in markers of bone erosion, and patients reported feeling better overall^[133-135]^.

An intriguing hypothesis suggests that the beneficial effects of the natural flavonoid resveratrol on arthritis may be mediated through its positive impact on the gut microbiome. Resveratrol is recognized for its anti-inflammatory properties and has demonstrated potential in alleviating RA symptoms in both animal and human studies^[136-138]^. A 2019 study suggested that resveratrol may influence intestinal microbiota, though it remains unclear whether this interaction contributes to its effects against RA. While many of these research avenues are still in early stages, the evidence gathered thus far could serve as a foundation for comprehensive double-blind randomized controlled trials exploring microbiota-focused dietary interventions for RA^[139,140]^.

### Inhibiting immune cell trafficking

Discussions regarding the movement of immune cells from the intestines to the joints suggest that strategically manipulating the migration of T effector cells from the gut to other areas of the body may provide a novel approach to treating inflammatory arthritis. For example, the retinoic acid analogue AM80 enhances the expression of the gut-homing molecule α4β7 integrin on T follicular helper (TFH) cells, effectively redirecting these cells away from inflamed regions of the intestine and thereby reducing arthritis severity in K/B × N mice^[141-143]^. Conversely, blocking β7 integrins resulted in increased arthritis severity in these mice; however, this effect was only observed in the presence of segmented filamentous bacteria (SFB). This interaction occurs because the blockade of β7 integrins, combined with SFB, promotes the expansion of α4β7+ TFH and α4β7+ TH17 cells, which accumulate at sites of inflammation due to their inability to return to the intestine, thereby worsening arthritis symptoms.

Given that alterations in intestinal barrier function are integral to the activation and migration of mucosal immune cells, it is noteworthy that enhancing the intestinal barrier during the early stages of arthritis using agents such as butyrate or a cannabinoid receptor 1 (CB1) agonist can slow disease progression^[144,145]^. Additionally, the role of zonulin in increasing intestinal permeability and potentially promoting the spread of inflammation to the joints is significant. Administration of larazotide acetate, a zonulin inhibitor, before the onset of arthritis in CIA mice resulted in a 50% reduction in arthritis severity. These findings suggest that targeting the molecular mechanisms governing intestinal immune cell migration - such as α4β7 integrin or zonulin - could represent a promising therapeutic strategy for RA. However, challenges for clinical application may include ensuring specificity to pathogenic immune cells, avoiding interference with normal immune surveillance, variability in patient microbiota that may influence efficacy, and potential off-target effects on other tissues^[146]^. This treatment improved intestinal barrier function and prevented the migration of activated intestinal cells to systemic organs and joints.

Moreover, the detection of CD11c+ CD103+ dendritic cells, normally present in the intestinal lamina propria, within the spleens of CIA mice indicates that these cells migrate from the gut to the spleen^[147-149]^. These experimental findings not only support the existence of a gut-joint axis in RA but also underscore its significance in disease progression, revealing several potential therapeutic targets^[150]^.

### Fecal microbiota transplantation

FMT involves transferring the gut microbiome from a healthy donor to another patient, aiming to restore the balance of the gut ecosystem. Since its recognition in 2013, FMT has been incorporated into professional guidelines as a standard treatment for recurrent and persistent *Clostridioides difficile *(*C. difficile*) infections^[151-153]^. Its application has expanded to include autoimmune diseases, driven by reports from patients with recurrent *C. difficile* infections who experienced unexpected improvements in celiac disease symptoms. Research using animal models of autoimmune disorders has demonstrated that FMT can mitigate gut imbalances, improve the function of autoreactive CD4+ T cells, and alleviate the severity of clinical symptoms. In human studies, FMT has been associated with a more balanced gut microbiota and increased production of beneficial SCFAs in individuals with multiple sclerosis and psoriatic arthritis^[154,155]^. However, effectiveness has not been consistent across all clinical trials. For instance, one study observed no significant improvement in a patient with Sjögren syndrome following FMT, while another reported only minimal enhancements in physical function for patients with psoriatic arthritis who underwent the procedure^[156,157]^.

Recent research has investigated the protective effects of FMT in RA. A supplement containing tuna oil was found to help restore gut microbiota balance and enhance the function of the intestinal epithelial barrier in mice with CIA. The altered gut microbiota from tuna oil-treated mice was subsequently used for FMT in control CIA mice. Further investigation into elastin peptides - derived from the breakdown of tuna elastin - revealed that transferring gut flora from mice treated with these peptides had therapeutic benefits^[158,159]^. This effect was characterized by reduced production of pro-inflammatory cytokines [IL-1β, Tumor necrosis factor alpha (TNF-α)] and increased levels of anti-inflammatory cytokines (IL-2, IL-10). However, classifying cytokines strictly as “pro-” or “anti-inflammatory” oversimplifies the highly dynamic and context-dependent nature of immune signaling, which varies according to disease stage, tissue microenvironment, and interactions with other immune and microbial cues^[160]^. After FMT, the gut microbiota was normalized at the phylum level, particularly with an increase in *Firmicutes* from 52.50% to 75.01%. Notably, at the genus level, there was a decrease in *Muribaculum* and a significant increase in *Lactobacillaceae*, from 31.51% to 59.16%, in mice that received FMT from those treated with tuna elastin peptides. Correlation analyses indicated a negative association between the abundance of *Prevotella sp*., *Helicobacter typhlonius*, and *Lentilactobacillus hilgardii *and levels of SCFAs such as valeric acid, butyric acid, and acetic acid^[160,161]^. In contrast, a positive correlation was noted between *Clostridium aerotolerans* and valeric acid production.

Certain studies have demonstrated promising results for the use of FMT in RA treatment, highlighting reductions in arthritis severity and RF levels in patients with refractory RA. However, careful screening of FMT donors is essential due to reported cases of infectious complications following treatment^[162,163]^. An evaluation of FMT long-term safety for patients with *C. difficile* infection confirmed its overall safety, despite one case indicating a potential increase in myocardial infarction risk. Additionally, a recent cross-sectional study found that individuals with prevalent autoimmune conditions such as RA, systemic lupus erythematosus, and psoriasis were less familiar with FMT compared to those with ulcerative colitis or Crohn disease (*P* < 0.001)^[164,165]^. Furthermore, general awareness of FMT was identified as the primary factor influencing acceptance of the treatment (*P* < 0.001), underscoring the urgent need for educational efforts by healthcare providers to improve patient understanding and address misconceptions about FMT^[166,167]^. The authors of these studies have proposed several explanations for why this approach did not yield the expected therapeutic effect, including insufficient microbial engraftment, high inter-individual variability in baseline microbiota composition, and the possibility that the targeted taxa do not play a causal role in disease progression^[166,167]^. Some studies also note that host immune activation or metabolic context may override microbiota-directed interventions, limiting their efficacy^[168]^.

Therefore, ongoing research in this area is essential.

## AUTOPHAGY REGULATION

Autophagy plays a crucial role in maintaining intestinal mucosal homeostasis through intestinal epithelial cells (IECs) and in regulating the balance of gut microbiota. Dysfunction in IEC autophagy has been identified as a significant factor in RA. Thus, enhancing autophagy in IECs may strengthen the intestinal barrier and optimize gut microbiota composition, presenting a potential therapeutic approach for early-stage RA^[169,170]^.

Research indicates that modifying gut microbiota alongside regulating autophagy can effectively reduce gut inflammation. For instance, altering the microbiota in mice deficient in the autophagy related 7 (Atg7) gene within IECs led to a marked increase in the presence of *Dubosiella* and *Turicibacter*, which correlated with more severe dextran sodium sulfate-induced colitis. In contrast, promoting autophagy has been shown to create a more balanced bacterial composition in the gut and prevent intestinal inflammation^[171,172]^. Preliminary studies have explored the protective effects of autophagy enhancers in an IBD mouse model. The addition of spermidine, a natural autophagy promoter, increased the abundance of Firmicutes in the gut and potentially influenced microbial functions related to amino acid, nucleotide, and lipid metabolism. This modulation was associated with positive outcomes, including enhanced expression of genes linked to tight junction integrity [Tight junction protein ZO-1 (Tjp1), Claudin-7 (Cldn7), Claudin-1 (Cldn1), and Tight junction protein ZO-3 (Tjp3)]^[173,174]^. Additionally, galangin, another natural autophagy enhancer, demonstrated protective effects against colon mucosal inflammation by promoting the growth of *Lactobacillaceae* and boosting SCFA production, particularly acetic and butyric acids^[175,176]^.

From a mechanistic standpoint, boosting autophagy in the gut may mitigate early inflammatory processes in individuals who are still in the preclinical phase of RA. This enhancement can help restore microbial balance, support the synthesis of anti-inflammatory metabolites, and strengthen epithelial barrier integrity^[177,178]^. Alterations in specific bacterial groups - most notably increases in *Bifidobacterium dentium* and members of *Verrucomicrobia* such as *Akkermansia* - appear capable of triggering autophagy through pathways involving toll-like receptor 4 (TLR4) and mechanistic target of rapamycin (mTOR). Activation of these mechanisms may, in turn, reduce intestinal inflammation by increasing mucus thickness, enhancing goblet cell activity, and inhibiting nuclear factor kappa-light-chain-enhancer of activated B cells (NF-κB) signaling, oxidative injury, and downstream cytokine production. As a result, therapeutic strategies that combine probiotic administration with microbial species known to directly induce autophagy in IECs may offer a means of slowing or preventing the transition from preclinical to clinical RA^[179-181]^. Nevertheless, emerging evidence indicates that excessive or dysregulated autophagy activation in the gut could drive M1 macrophage polarization, a process potentially intensified by shifts in the microbiome and by Paneth cell metaplasia. Such outcomes raise the concern that high autophagy levels - particularly in individuals exposed to substantial psychosocial stress - may aggravate IBD rather than provide protection^[182,183]^.

Given the complexity of the downstream pathways in IEC autophagy and the intricate interactions between autophagy and the gut microbiome, extensive research is needed to assess the potential benefits and drawbacks of moderating IEC autophagy as a strategy for treating preclinical RA. Furthermore, enhancing the screening processes for autophagy inducers is essential to identify those that provide optimal anti-inflammatory effects while curbing RA progression and minimizing the risk of triggering IBD^[184-186]^.

## THE CASE OF MIRNAS

Recent studies suggest that miRNAs may play a role in the onset of early RA. Several miRNAs, including miRNA-449, miRNA-27b-3p, miRNA-495, miRNA-34a-3p, miRNA-340-5p, and miRNA-17-5p, have been shown to inhibit the proliferation of RA synovial fibroblasts by targeting the signal transducer and activator of transcription 3 (STAT3)/β-catenin signaling pathway, reducing the expression of cell cycle proteins, and decreasing histone deacetylase 1 production^[187-189]^. Despite these findings, research on miRNA-based therapies for pre-clinical RA is still in its early stages. However, insights from treatments for other dysbiosis-related diseases may inform future strategies for managing pre-clinical RA. In the context of IBD, studies investigating the use of exogenous miRNAs have shown promising results, indicating that miRNA treatments can reduce intestinal inflammation by modifying gut microbiota composition and modulating the intestinal immune response^[190,191]^.

In experimental colitis models receiving mesenchymal stem cell-derived miRNAs, investigators observed distinct shifts in gut microbial structure. Treatment with miRNA-181a led to increased alpha diversity, reflected in higher values of observed operational taxonomic units, Chao1, and the Abundance-based Coverage Estimator^[192,193]^. At the genus level, administration of the miRNA elevated the relative abundance of *Enterorhabdus, Lactobacillaceae*, and *Akkermansia*, while reducing *Bacteroides*. Family-level profiling further showed that miRNA-181a promoted expansion of *Lactobacillaceae* and *Bacteroidales* S24-7, accompanied by a decline in *Clostridiaceae.* Immunologically, treatment enhanced the proportion of Interleukin-10 (IL-10^+^) forkhead box P3 (Foxp3^+^) Tregs and lowered the frequency of Th17 cells within the intestinal mucosa, along with broad suppression of pro-inflammatory mediators such as IL-1β, IL-6, IL-2, IL-18, IL-17, and TNF-α. Conversely, enrichment of taxa including *Enterococcus, Turicibacter*, *Helicobacter, Desulfovibrionaceae*, unclassified *Desulfovibrionaceae*, and *Mogibacteriaceae* showed positive associations with elevated inflammatory cytokine levels and activation of immune-inflammatory pathways. Collectively, these results underscore the capacity of miRNAs to modulate gut microbial communities and thereby help preserve intestinal immune homeostasis^[194,195]^.

Conversely, some researchers propose that miRNAs may help foster an anti-inflammatory state and enhance the integrity of the gut barrier by directly interacting with the ycnE gene of *L. rhamnosus *monooxygenase, which is believed to stimulate the production of IL-22 - an important molecule for gut health^[196-198]^. IL-22 aids in the regeneration of the intestinal epithelium, enhances the expression of chemokines and cytokines for pathogen clearance, and promotes the growth of goblet cells. This theory was supported by observations that the ycnE gene was suppressed following treatment with ginger-derived mdo-miR7267-3p, resulting in a significant decrease in indole-3 acetamide (I3AM), a compound that inhibits the precursor to indole-3-carboxaldehyde (I3CA), ultimately increasing I3CA production^[199,200]^. Interestingly, IL-22 levels remained unchanged in aryl hydrocarbon receptor (AhR)-deficient mice treated with I3AM, underscoring the crucial role of AhR in suppressing the *L. rhamnosus* I3CA-induced IL-22 expression by I3AM. Thus, miRNAs could serve as effective mechanisms for preserving intestinal barrier function and regulating immune responses by modulating metabolites produced by specific gut bacteria^[201,202]^.

miRNAs also contribute to the preservation of gut barrier function by reinforcing epithelial architecture. In mouse models of IBD, marked upregulation of miRNA-602 resulted in clear symptomatic improvement - most notably reduced diarrhea—and was accompanied by elevated expression of barrier-supporting genes such as Zonula Occludens-1 (ZO-1), Mucine-2 (MUC2), and MUC3^[203,204]^. Consistent with these findings, treatment of colonic injury with mesenchymal stem cell-derived miRNA-181a restored the expression of key tight junction components, including claudin-1 and ZO-1. Moreover, plant-derived miRNAs provide another layer of protection: ginger-associated miRNAs were shown to suppress both transcription and protein levels of the *L. rhamnosus* pilus adhesin SpaC, thereby limiting the organism’s ability to accumulate within the mucosal layer. These observations suggest that host or dietary miRNAs can directly modulate bacterial behavior, contributing to enhanced resistance against pathogenic or excessive microbial colonization^[205,206]^.

Overall, the findings indicate that altering gut microbiota using miRNAs could be a promising strategy to enhance the stability of the microbial symbiont community, reduce intestinal inflammation, and improve gut barrier function in individuals with pre-clinical RA. Further clinical research is essential to assess the effectiveness and safety of miRNA-based therapies and to provide additional evidence regarding the benefits of this approach^[207-209]^.

## FUTURE PERSPECTIVES

Dysbiosis of the gut microbiota occurs during the pre-clinical stage of RA and significantly contributes to the disease development. Various mechanisms have been identified that link microbiota imbalance with RA progression, including molecular mimicry, the effects of microbiome-produced metabolites, impaired intestinal barrier function, immune responses triggered by the microbiome, autophagy in IECs, and alterations in miRNA expression^[210,211]^.

Current RA treatments are typically initiated only after the disease has manifested, and their long-term use can result in numerous side effects. Therefore, addressing gut microbiota during the pre-clinical stage may represent a considerable advancement in RA management. Interventions such as dietary modifications and prebiotics - including a high-fiber diet, fish oil supplementation, and inulin consumption - have the potential to correct microbiota imbalances and restore immune balance in individuals at this early stage of RA^[212]^. However, extensive research in pre-clinical populations is essential to confirm the effectiveness of these strategies in preventing arthritis onset. While oral antibiotics may alleviate arthritis symptoms, their preventive use could disrupt the delicate balance of the intestinal microbiota and potentially trigger RA development. Moreover, FMT has shown promise in restoring intestinal bacterial balance and barrier function in CIA models, but applying FMT before arthritis onset poses risks, including potential infections and further disruption of gut microbiota^[213]^.

Innovative strategies for managing gut microbiota in the early stages of RA are emerging, including the use of probiotics, natural compounds, autophagy modulation, miRNA therapies, and vitamin D supplementation. Natural compounds commonly used in RA treatment can effectively alleviate gut dysbiosis while maintaining safety. Furthermore, optimizing gut microbiota could enhance the efficacy of these natural compounds in slowing RA progression and influencing immune cell behavior. Modulating autophagy in IECs has the potential to rebalance gut microbiota and restore intestinal barrier integrity, thereby preventing early RA advancement, though further research is warranted in this area^[214]^. The ability of miRNAs and vitamin D to influence microbiota highlights their potential roles in preventing early-stage RA. Previous studies have indicated that probiotics can modify gut microbiota and provide symptomatic relief from RA. Given the strong preventive effects of probiotics on RA development demonstrated in animal studies, further investigation into their application for gut microbiota management in early RA appears promising^[215]^. Additionally, combining probiotics with dietary modifications could enhance preventive measures against RA. Therefore, a comprehensive approach that incorporates probiotics, dietary changes, natural compounds, and vitamin D therapy to manage microbiota and prevent RA progression is a valuable avenue for future research.

This summary underscores groundbreaking insights into how changes in microbiota during early RA can enhance our understanding of immune dysfunctions at this stage. Several potential therapeutic strategies have been identified that could ultimately aid in preventing RA progression and improving disease remission during the pre-clinical phase^[216]^.

## CONCLUSION

The strong connection between gut microbiota and the onset of RA highlights the urgent need for early intervention strategies targeting the preclinical phase of the disease. As emerging evidence increasingly points to dysbiosis as a key factor in RA development, it becomes evident that understanding the dynamics of microbiota offers a promising path for both prevention and treatment. Current therapeutic approaches primarily focus on managing established disease, which often results in adverse side effects. In contrast, targeting gut microbiota through dietary modifications, probiotics, and other microbiome-centered strategies may not only restore balance but also reduce inflammation and potentially halt RA progression.

Research into specific microbial species such as *P. copri* and *Collinsella*, along with advancements in therapies such as FMT and autophagy modulation, presents exciting opportunities for intervention. Furthermore, investigating miRNA modulation offers valuable insights into the complex relationship between gut health and immune function. However, additional clinical studies are necessary to validate these strategies and assess their effectiveness in preclinical populations.

In conclusion, enhancing our understanding of the gut-joint axis creates new opportunities for RA prevention. A proactive, multifaceted approach that combines nutritional, microbial, and immunological insights will be essential for transforming therapeutic paradigms, ultimately leading to better outcomes for individuals at risk for RA.

## DECLARATIONS

### Copyright

© The Author(s) 2026.
